# Effects of ω-3 PUFA-Rich Oil Supplementation on Cardiovascular Morphology and Aortic Vascular Reactivity of Adult Male Rats Submitted to an Hypercholesterolemic Diet

**DOI:** 10.3390/biology11020202

**Published:** 2022-01-27

**Authors:** Mariely Mendes Furtado, Joana Érica Lima Rocha, Ana Victória da Silva Mendes, Renato Sampaio Mello Neto, Ana Karolinne da Silva Brito, José Otávio Carvalho Sena de Almeida, Emerson Iuri Rodrigues Queiroz, José Vinícius de Sousa França, Ana Lina de Carvalho Cunha Sales, Andreanne Gomes Vasconcelos, Wanessa Felix Cabral, Luana de Oliveira Lopes, Iolanda Souza do Carmo, Selma Aparecida Souza Kückelhaus, José Roberto de Souza de Almeida Leite, Adriana Maria Viana Nunes, Marcia dos Santos Rizzo, Antônia Maria das Graças Lopes Citó, Ana Karina Marques Fortes Lustosa, Massimo Lucarini, Alessandra Durazzo, Maria do Carmo de Carvalho e Martins, Daniel Dias Rufino Arcanjo

**Affiliations:** 1 Department of Biophysics and Physiology, Federal University of Piauí, Teresina 64049-550, PI, Brazil; marielymf@live.com (M.M.F.); ericarocha6@hotmail.com (J.É.L.R.); victoriams18@hotmail.com (A.V.d.S.M.); renato.sampaio.mn@gmail.com (R.S.M.N.); anakarolinnesb@hotmail.com (A.K.d.S.B.); otavios.almeida@hotmail.com (J.O.C.S.d.A.); emersoniuri@ufpi.edu.br (E.I.R.Q.); vinicius.sfranca@ufpi.edu.br (J.V.d.S.F.); ana.lina123@gmail.com (A.L.d.C.C.S.); adriananunes@ufpi.edu.br (A.M.V.N.); carminhamartins@ufpi.edu.br (M.d.C.d.C.e.M.); 2 University Hospital, Federal University of Piauí, Teresina 64049-550, PI, Brazil; 3 Research Center in Morphology and Applied Immunology, Faculty of Medicine, University of Brasília, Brasília 70910-900, DF, Brazil; andreannegv@gmail.com (A.G.V.); wanessa.felix@unb.br (W.F.C.); luanalopes.ufpi@gmail.com (L.d.O.L.); selmask@gmail.com (S.A.S.K.); jrsaleite@gmail.com (J.R.d.S.d.A.L.); 4 Department of Chemistry, Federal University of Piauí, Teresina 64049-550, PI, Brazil; iolandasouzadocarmo@outlook.com (I.S.d.C.); gracito@ufpi.edu.br (A.M.d.G.L.C.); 5 Department of Morphology, Federal University of Piauí, Teresina 64049-550, PI, Brazil; marciarizzo@ufpi.edu.br; 6 Galeno Farmácia de Manipulação, Virgínia Regina Fortes Castelo Branco e Cia. Ltd.a., Teresina 64001-260, PI, Brazil; ana_lustosa@uol.com.br; 7 CREA-Research Centre for Food and Nutrition, Via Ardeatina 546, 00178 Rome, Italy; massimo.lucarini@crea.gov.it (M.L.); alessandra.durazzo@crea.gov.it (A.D.)

**Keywords:** cardiovascular diseases, atherosclerosis, endothelium, hypercholesterolemia, fatty acids, omega-3, fish oil, dietary supplements

## Abstract

**Simple Summary:**

Currently, processed and ultraprocessed foods represent a significant component of the diet of modern societies, increasing the risk of developing obesity, diabetes and atherosclerosis. Therefore, replacing saturated fats with mono- and polyunsaturated fats, such as omega-3 polyunsaturated fatty acids (ω-3 PUFAs), has been considered as a dietary strategy to reduce clinical events related to atherosclerosis. In the present study, the effects of 56-day ω-3 PUFA-rich oil supplementation on liver function, lipid profile, and oxidative stress in hypercholesterolemic rats were investigated, as well as its impact on cardiovascular health. Interestingly, we observed a positive effect in reducing hepatic markers, preserving cardiovascular morphology, and increasing vasodilator responsiveness. These findings contribute to the generation of consistent recommendations for the therapeutic use of ω-3 PUFAs in the treatment of atherosclerosis, leading to a consequent reduction in related morbidity and mortality.

**Abstract:**

Atherosclerosis is a cardiovascular disease associated with abnormalities of vascular functions. The consumption of mono- and polyunsaturated fatty acids can be considered a strategy to reduce clinical events related to atherosclerosis. In the present study, we investigated the effects of supplementation with 310 mg of ω-3 PUFAs (2:1 eicosapentaenoic/docosahexaenoic acids) for 56 days on rats with hypercholesterolemia induced by a diet containing cholesterol (0.1%), cholic acid (0.5%), and egg yolk. Serum biochemical parameters were determined by the enzymatic colorimetric method. Assessment of vascular effects was performed by analysis of histological sections of the heart and aortic arch stained with hematoxylin and eosin and vascular reactivity of the aorta artery. We observed that treatment with ω-3 PUFAs did not promote alterations in lipid profile. On the other hand, we documented a favorable reduction in liver biomarkers, as well as contributions to the preservation of heart and aortic arch morphologies. Interestingly, the vascular reactivity of rat thoracic aortic preparations was improved after treatment with ω-3 PUFAs, with a decrease in hyperreactivity to phenylephrine and increased vasorelaxation promoted by acetylcholine. Our findings suggest that the supplementation of hypercholesterolemic rats with ω-3 PUFAs promoted improvement in liver and vascular endothelial function as well as preserving heart and aortic tissue, reinforcing the early health benefits of ω-3 PUFAs in the development of atherosclerotic plaque and further related events.

## 1. Introduction

Cardiovascular diseases (CVDs) represent a group of heart and blood vessel diseases that are the leading cause of death worldwide. From every five deaths from CVD, four are due to heart attacks and strokes, and a third of these deaths occur prematurely in people under 70 years old. Occurrence of heart attacks and stroke is usually due to a combination of risk factors, such as use of tobacco, obesity and consumption of a high-fat diet, sedentary lifestyle, harmful use of alcohol, systemic arterial hypertension (SAH), type 1 and 2 diabetes, and hyperlipidemia [1]. 

Hyperlipidemia, an augment to plasma lipid concentration, contributes to increased risk for CVD, which is closely associated with endothelial dysfunction, leading to an inflammatory response and oxidative stress and then to the development of atherosclerosis. The presence of these risk factors influences blood circulation, tissue oxygen distribution, and inflammation. Decreased nitric oxide (NO) bioavailability, increased reactive oxygen and nitrogen species (ROS and RNS), and increased endothelial dysfunction also underlie impaired vasodilator capacity [2,3]. 

In this context, atherosclerosis is the most important among CVDs [4,5,6], with great repercussions on morbidity and mortality and high socioeconomic impact. Atherosclerosis is characterized as a diffuse and degenerative inflammatory disease of the arteries with the formation of plaque consisting of necrotic cells, lipids, and cholesterol crystals [7]. Such plaques can cause stenosis, embolization, and distal thrombosis [6]. Atherosclerosis is associated with early abnormalities of vascular functions characterized by an attenuation of endothelium-dependent relaxation even before histological evidence of plaque formation [8].

Currently, processed and ultraprocessed foods represent a significant component of the diet of modern societies, leading to increased intake of lipids (saturated fats and cholesterol), the harmful effects of which on human health are already well established. Replacing saturated fat in the diet with mono- and polyunsaturated fat is considered a strategy for better control of hypercholesterolemia and consequent reduction in the occurrence of clinical events related to atherosclerosis [9,10]. 

Among the unsaturated fatty acids used to reduce the risk of CVD, ω-3 PUFAs have been associated with benefits in treatment of CVDs and have attracted attention because of their effects. As early as 1944, Sinclair learned of the rarity of coronary heart disease in Greenland’s Eskimos, who ate a diet rich in whales, seals, and fish. Thereafter, researchers from observational population studies of Greenland and Okinawans reported that the lower risk of death from coronary artery disease in natives compared with that in whites was related to the source of the PUFA ω-3 in their diet [11,12]. According to Mori, there is considerable evidence that these ω-3 PUFAs provide protection against CVD, positively affecting arterial pressure and compliance, vascular reactivity, and the lipid profile [13]. 

Recent studies have supported the antiatherogenic effects of ω-3 PUFAs [14,15]. Guidelines for CVD prevention in Brazil and worldwide have encouraged the consumption of foods containing ω-3 PUFAs daily as part of healthy eating [16]. However, despite the evidence, the recommendation to consume ω-3 PUFAs is controversial, and the discussion about their effects on early events that trigger CVD is relevant. Therefore, the present study aimed at evaluating the effects of supplementation with omega-3 polyunsaturated fatty acids on lipid profile biomarkers and oxidative stress, as well as evaluating the impact on vascular morphology and function, in a model of endothelial dysfunction induced by hypercholesterolemic diet in rats.

## 2. Materials and Methods

### 2.1. Analysis of the Composition of Omega-3 Polyunsaturated Fatty Acids

Initially, methyl derivatives of triacylglycerols were obtained from ω-3 polyunsaturated fatty acid-rich oil capsules (Catalent, Inc., Sorocaba, SP, Brazil; Batch No. 100001748), following the methodology described by Hartman and Lago (1973) [17]. Analyses of the methyl derivatives were carried out by gas chromatography coupled to a mass spectrometer, using a Shimadzu GC-17A/MS-QP505A instrument. Identification was made from a comparison with the fragmentation pattern available in the WILEY^®^ library and with others described in the literature.

### 2.2. Animals

Seventy-two male Wistar rats (6 months; 300–350 g) from the Central Animal Facility of UFPI were used. During the experimental period, the animals were kept in collective cages with four animals per cage, under controlled temperature (23 ± 2 °C), a 12 h light–dark cycle, and free access to food and water. The research was approved by the Ethics Committee on Animal Use at UFPI (Approval No. 446/18).

### 2.3. Experimental Diet

The planning of the experimental diet was based on the composition of the feed for animals in the maintenance phase with the addition of inducers for hypercholesterolemia, in accordance with other studies that induced dyslipidemia in rodents [18,19,20,21,22]. 

During the adaptation phase and for the animals in the standard control group, a commercial feed (Presence^®^) for rats and mice was used. The feed used to induce hypercholesterolemia was prepared manually, mixing the commercially ground feed with cholesterol (0.1%), cholic acid (0.5%), and egg yolk (4 units/100 g of feed). This mix was molded into pellets and dried in an oven at a temperature of 60 °C for approximately 24 h. After preparation, the hypercholesterolemic feed was kept under refrigeration (4 °C) until consumption.

### 2.4. Experimental Model

**Figure 1:**
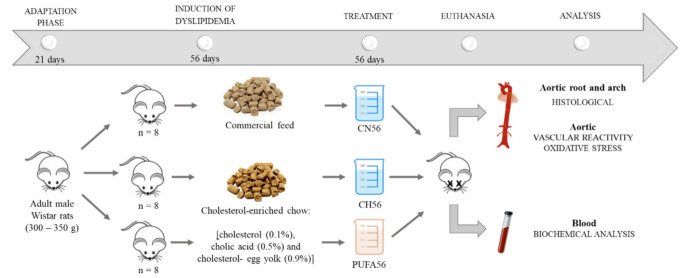
Experimental design of induction of dyslipidemia and treatment with ω-3 PUFA-rich oil.

The animals were randomly divided into three groups (n = 8/group). In one of the groups (CN56), the animals received standard rodent chow, and in the other two groups (CH56; PUFA56) the cholesterol-enriched chow was used for eight weeks before starting treatment. After eight weeks for induction of dyslipidemia, supplementation with omega-3 PUFA was started in the PUFA56 group. The treatment was administered once a day. The content of the omega-3 capsules (1 mL) was aspirated and administered orally (gavage). During the treatment period, the animals were fed the same type of diet used since the beginning of the experiment (Figure 1). This experimental design and all analyses were performed in triplicate with N = 8 per group in each repetition.

At the end of the treatments, blood was collected to obtain serum for biochemical analysis. The aorta was removed through a midline chest incision and fragments were collected for analysis of oxidative stress and antioxidant enzymes and assessment of vascular reactivity. The aortic root and arch were used for histological processing. 

### 2.5. Evaluation of the Effects of ω-3 Fatty Acids on Serum Biochemical Parameters

Serum levels of albumin, total proteins, alanine aminotransferase (ALT), aspartate aminotransferase (AST), total cholesterol (CT), HDL cholesterol, and triglycerides (TG) were determined by the enzymatic colorimetric method in an automatic analyzer and commercial kits according to the methodology recommended by the manufacturer (Labmax Plenno, Labtest, Lagoa Santa, MG, Brazil). LDL cholesterol was calculated according to Friedewald’s formula [23] as follows: LDL = CT − HDL − VLDL. The TG/5 value is an estimate of VLDL, and all concentrations were expressed in mg/dL.

### 2.6. Evaluation of the Effects of ω-3 Fatty Acids on Histopathological Changes in Heart and Aortic Arch Segments

The heart and aortic arch fragments were fixed for 24 h in a glycerin formaldehyde solution and transferred to 70% alcohol. The material was processed using histotechnical equipment Mod 808 (ANCAP Equipamentos Eletroeletrônicos LTDA, São Paulo, Brazil). After impregnation and immobilization in paraffin, the fragments were sectioned at a thickness of 5 μm and stained with hematoxylin and eosin (HE). The slides were scanned using a 40× objective on a Scan Scope Aperio (Leica Biosystem Inc., Lincolnshire, IL, USA) equipped with a Scan Scope Cansole v.10.2.0.2352 image capture and processing system. Histopathological analysis was performed blindly in all histological sections, evaluating the general architecture of the tissues in a qualitative way, using the Aperio Image Scope software version 12.4.0.5043 (Leica Biosystem Inc., USA).

### 2.7. Ex Vivo Assessment of Vascular Effects of ω-3 Fatty Acids after Supplementation

The thoracic portion of the aorta artery was dissected, with removal of perivascular tissue that was subsequently sectioned into rings approximately 3 mm in length and kept in 10 mL vats for isolated organs containing Krebs solution (in mM: NaCl, 118.0; KCl, 4.6; CaCl_2_·2H_2_O, 2.5; MgSO_4_·7H_2_O, 5.7; NaHCO_3_, 25.0; KH_2_PO_4_·H_2_O, 1.1; and D-glucose, 11.0) under tension of 1.0 g, a temperature of 37 °C, and aerated with a mixture of CO_2_ (5%), O_2_ (21%), and N_2_ (74%) [24].

The rings were suspended by cotton threads and fixed to force transducers coupled to a data acquisition system for recording isometric tensions (AQCAD 2.3.8., AVS Projetos, SP, Brazil). After 45 min of stabilization, concentration–response curves were obtained with cumulative concentrations (10^−9^ to 10^−5^ M) of phenylephrine (FEN), a selective α1-receptor antagonist that promotes vasoconstriction, as well as cumulative concentrations (10^−9^ to 10^−5^ M) of acetylcholine (ACh) or sodium nitroprusside (NPS) (endothelium-dependent and -independent vasodilators, respectively) in FEN-precontracted aortic preparations.

The results for each concentration are expressed as a percentage of the maximum contraction obtained by FEN or the maximum relaxation obtained by ACh and NPS. The results are expressed by the values of pD_2_ (negative logarithm of the EC_50_) and E_max_ (maximum effect) calculated from nonlinear regression of the concentration–response curves.

After obtaining aortic artery ring preparations, a standard concentration–response curve for ACh (10^−9^–10^−5^ M) was obtained. The preparations were washed with Krebs solution, and after stabilization, they were preincubated for 30 min with pyrogallol (30 µM), a superoxide anion donor. Then, the preparations were precontracted with phenylephrine (10 µM), and after 20 min, cumulative concentrations of ACh (10^−9^–10^−5^ M) were added.

### 2.8. Assessment of the Effect of ω-3 Fatty Acids on Oxidative Stress and Antioxidant Defenses in Aortic Artery (MPO, NO_2_^−^, SOD and CAT)

The aortic fragment was quickly removed and homogenized (1:20 *w*/*v*) in phosphate buffer with pH adjusted according to the analysis to be performed. The measurement of myeloperoxidase (MPO) activity was based on the rate of formation of the oxidation product of o-dianisidine in the presence of H_2_O_2_ and performed by observing the increase in the absorbance of the mixture at 450 nm [25]. The concentration of nitrite (NO_2_^−^) was determined by the Griess method as described by Green et al. (1982) [26]. The measurement of superoxide dismutase (SOD) activity was based on the ability to consume the O_2_**^−^** radical, decreasing the autoxidation ratio of pyrogallol (1,2,3-trihydroxybenzene) [27]. Catalase activity (CAT) was determined according to the method described by Beutler (1975) [28].

### 2.9. Statistical Analysis

Data are represented as mean ± standard error of the mean (SEM). Statistical analysis was performed by applying the unpaired Student’s *t*-test or two-way analysis of variance (ANOVA) followed by the Bonferroni posttest, when applicable. For all statistical analyses, pD_2_ calculations, EC_50_, and curve plotting, the statistical software GraphPad Prism 8.0 (Graph Pad Software, Inc., La Jolla, CA, USA) was used. The level of significance established was *p* < 0.05.

## 3. Results

The topic is explored as follows: (i) identification of fatty acid methyl derivatives by gas chromatography coupled with a mass spectrometer (GC–MS); (ii) effect of ω-3 PUFA-rich oil on serum biochemical parameters; (iii) effect of ω-3 PUFA-rich oil on histological changes in heart and aortic arch segments; (iv) effects of ω-3 PUFA-rich oil on vascular reactivity of rat thoracic aorta; (v) effects of ω-3 PUFA-rich oil on oxidative stress and antioxidant defenses in rat aortic tissue.

### 3.1. Identification of Fatty Acid Methyl Derivatives by Gas Chromatography Coupled with a Mass Spectrometer (GC–MS)

**Figure 2:**
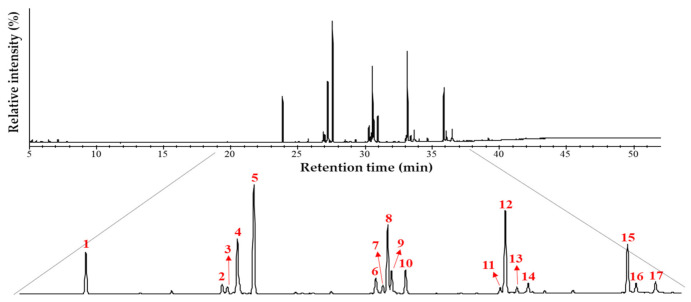
Total ion chromatogram (TIC) of methyl esters derived from ω-3 polyunsaturated fatty acids. Legend: x-axis: ‘Retention time (min)’; y-axis: ‘Relative intensity (%)’.

The chromatograms of fatty acid methyl derivatives were obtained by GC–MS (Figure 2), and the identity and relative intensity (%) of fatty acids were determined (Table 1). Nineteen fatty acids from the capsules were identified, representing 98.47% from detected constituents. The n-6/n-3 ratio was approximately 1:9. Among ω-3 PUFAs, the ratio between DHA and EPA, the major ω-3 PUFAs identified, was close to 1:2 (Table 1). 

### 3.2. Effect of ω-3 PUFA-Rich Oil on Serum Biochemical Parameters

The consumption of hypercholesterolemic feed promoted increases in serum total cholesterol (*p* = 0.0172), LDL cholesterol (*p* = 0.0037), and glucose (*p* = 0.0491) in the animals of the CH56 group when compared to those in the CN56 group. On the other hand, no differences regarding the levels of TG (*p* = 0.2151), VLDL (*p* = 0.1822), or HDL cholesterol were found after the consumption of hypercholesterolemic feed. The treatment of hypercholesterolemic rats with ω-3 PUFA-rich oil for 56 days did not change any of these parameters when compared with the CH56 group (Table 2). 

The serum levels of total proteins and alanine aminotransferase (ALT) were increased in the CH56 group when compared to the CN56 group (*p* < 0.05). Animals treated with ω-3 PUFA-rich oil for 56 days presented significantly lower serum albumin, total protein, alanine aminotransferase (ALT), and aspartate aminotransferase (AST) (*p* < 0.05) than those in the CH56 group (Table 2).

### 3.3. Effect of ω-3 Fatty Acids on Histological Changes in Heart and Aortic Arch Segments

**Figure 3:**
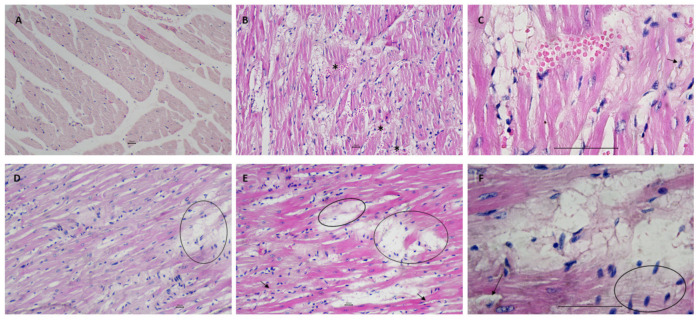
Photomicrographs of myocardial tissue from the CN56 group (**A**), CH56 group (**B**,**C**), and PUFA56 group (**D**–**F**). Hematoxylin–eosin (HE) stain (scale bar: 20 µm and 60 µm). (**A**): Rounded cross-sections of cardiac muscle cells with a centrally located nucleus. (**B**): Longitudinal section of myofiber disarray, with diffuse myodegeneration and hemorrhagic areas (*). (**C**): Detail of B showing foam cell (arrow). (**D**): Myofibers with a branched appearance, some showing vacuolar degeneration (circle) and infiltration of mononuclear cells. (**E**): Numerous mononuclear cells and fibroblasts, capillaries (arrows), and vacuolar degeneration areas (circle). (**F**): Detail of E showing foam cell (arrow) and fibroblasts (circle).

Figure 3A shows, for the CN56 group, normal integrity of myocardial tissue with striations with a centrally located nucleus. On the other hand, animals from the CH56 group presented diffuse myodegeneration with significant cytoplasmic vacuolization, myofiber disarray, multifocal hemorrhagic areas, and infiltration of mononuclear cells with multiple foam cells (Figure 3B,C). Interestingly, the analysis of the PUFA56 group showed some foci of myofiber disarrangement and an absence of hemorrhagic areas (Figure 3D–F). A large part of cardiac tissue had myofibers with a branched appearance, some of which showed vacuolar degeneration. Furthermore, moderate infiltration of mononuclear cells and some foam cells, capillaries, and fibroblastic proliferation were observed.

**Figure 4:**
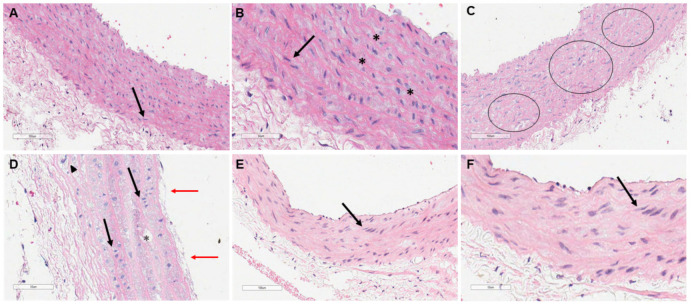
Photomicrographs of aorta from CN56 group (**A**,**B**), CH56 group (**C**,**D**), and PUFA56 group (**E**,**F**). Hematoxylin–eosin (HE) stain (scale bar: 100 µm and 50 µm). (**A**): This section shows the tunica media, composed of alternating layers of circumferentially arranged smooth muscle cells (arrow). (**B**): Detail of (**A**) showing wavy sheets of elastic fibers (*) and smooth muscle cells (arrow). (**C**): This section shows the disarrangement of the elastic fibers and the vacuolization of smooth muscle cells (circles) of the middle layer of the aorta. (**D**): Detail of (**C**) showing extracellular lipid (*), smooth muscle cell proliferation (black arrows), foam cell (arrowhead), and epithelial denudation (red arrows). (**E**,**F**): Note the reorganization of the histological structure of the artery, with more evident elastic fibers and a decrease in the vacuolar aspect of the smooth muscle cells (arrows).

Figure 4 shows aortic arch microscopy representing one animal from each experimental group (CN56, CH56, and PUFA56). In the CN56 group, the tunica intima was very thin and could not be distinguished from the tunica media, which displayed a series of concentrically organized elastic laminae (Figure 4A,B) in which vascular smooth muscle cells (VSMCs) were well preserved. On the other hand, a structural disarrangement of elastic fibers in the tunica media was observed in the CH56 group, with VSMCs presenting vacuolated cytoplasm, denoting a ‘foamy’ appearance (Figure 4C). The VSMCs also presented rounded and row nuclei (Figure 4D), which indicated proliferative activity and a consequent increase in collagen deposition. This was confirmed by Masson’s trichrome stain (see Figure S1). In the tunica intima, adhesion of some leukocytes and a fibrin network to the endothelium was observed, with areas of slight subendothelial thickening (Figure 4D).

Interestingly, the aortic arches of animals supplemented with ω-3 PUFA-rich oil presented very thin, preserved tunicae intimae similar to observed in CN56 animals’ aortic arches (Figure 4E,F). In the tunica media, a reorganization of the histological structure of the vessel was observed, with more evident elastic fibers and a decrease in the vacuolar aspect of the VSMCs (Figure 4E).

### 3.4. Effects of ω-3 PUFA-Rich Oil on Ex Vivo Vascular Reactivity of Rat Thoracic Aorta 

**Figure 5:**
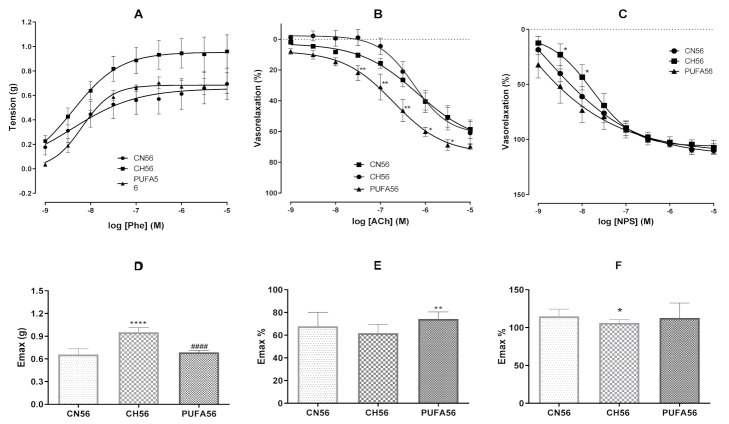
Concentration–response curves induced by phenylephrine (**A**,**D**), acetylcholine (**B**,**E**) and sodium nitroprusside (**C**,**F**) in aortic artery rings from hypercholesterolemic rats treated with ω-3 PUFA-rich oil for 56 days, and Emax values thereof. Results are expressed as mean ± SEM (n = 6–10). Unpaired Student’s *t*-test. * *p* < 0.05, ** *p* < 0.01 and **** *p* < 0.0001 vs. CN56; #### *p* < 0.0001 vs. CH56.

In aortic artery preparations, the phenylephrine (FEN)-induced vasoconstrictor response was increased in hypercholesterolemic animals from the CH56 group (E_max_ = 0.952 ± 0.063 gf) when compared with animals from the CN56 group (E_max_ = 0.6567 ± 0.0782 gf). Interestingly, this vasoconstrictor response was markedly attenuated after treatment with ω-3 PUFA-rich oil for 56 days (E_max_ = 0.687 ± 0.024 gf) (Figure 5A,D). 

As marked evidence of improvement in vascular endothelial function after treatment with ω-3 PUFA-rich oil for 56 days, an increase in acetylcholine (ACh)-induced vasorelaxation in aortic artery preparations was observed (Emax = 74.20 ± 6.24%) when compared with preparations from the CH56 group (Emax = 61.73 ± 7.77%) (Figure 5B,E). On the other hand, regarding endothelium-independent vasorelaxation, treatment with ω-3 PUFA-rich oil for 56 days potentiated the sodium nitroprusside (NPS)-induced vasorelaxation, with no significant alterations in Emax when compared with CH56 group (Figure 5C,F). 

**Figure 6:**
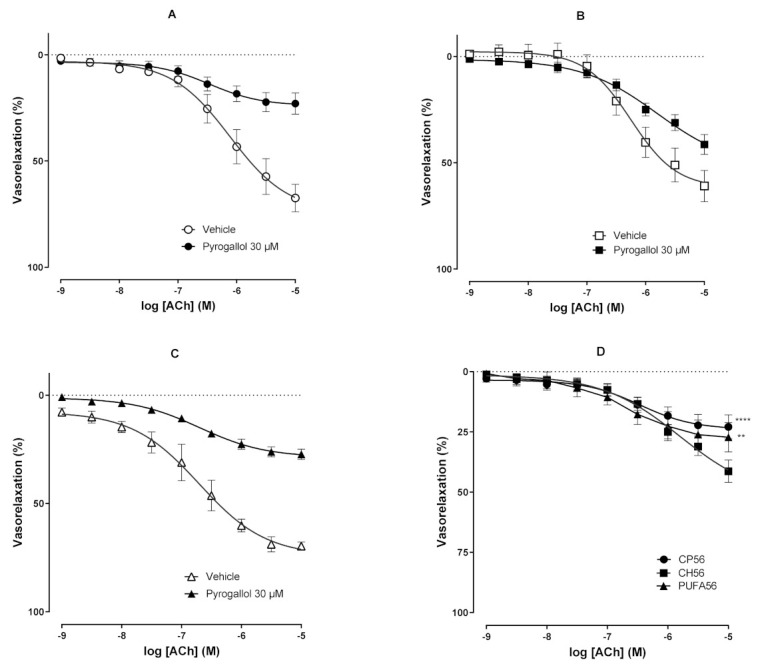
Concentration–response curves induced by acetylcholine (10^−9^–10^−5^ M) in the absence or presence of pyrogallol (30 µM) in aortic artery rings from hypercholesterolemic rats treated with ω-3 PUFA-rich oil for 56 days. Results are expressed as mean ± SEM. Two-way ANOVA followed by Bonferroni posttest. ** *p* < 0.01 vs. CN56; **** *p* < 0.0001 vs. CH56. (**A**) CN56; (**B**) CH56; (**C**) PUFA56; (**D**) concentration–response curves for acetylcholine in the presence of pyrogallol.

In another set of experiments, ex vivo aortic preparations from the CN56, CH56, and PUFA56 groups were pretreated with pyrogallol (30 µM) and then precontracted with phenylephrine, followed by an obtention of the acetylcholine concentration–response vasorelaxant curve. The acetylcholine-induced vasorelaxant effect was significantly reduced in all groups, as an expected pyrogallol-induced vascular effect (Figure 6A–C). Interestingly, in aortic artery preparations from hypercholesterolemic animals supplemented with ω-3 PUFA-rich oil for 56 days (PUFA56 group), the acetylcholine-induced concentration–response curve was significantly attenuated when compared with that of animals from the CH56 group. This finding indicates a vasoprotective effect induced by the treatment with ω-3 PUFA-rich oil for 56 days in hypercholesteremic rats via restoring the acetylcholine-induced vasoactive effect to levels comparable to those in normal rats (Figure 6D).

### 3.5. Effects of ω-3 PUFA-Rich Oil on Oxidative Stress and Antioxidant Defenses in Rat Aortic Tissue

**Figure 7:**
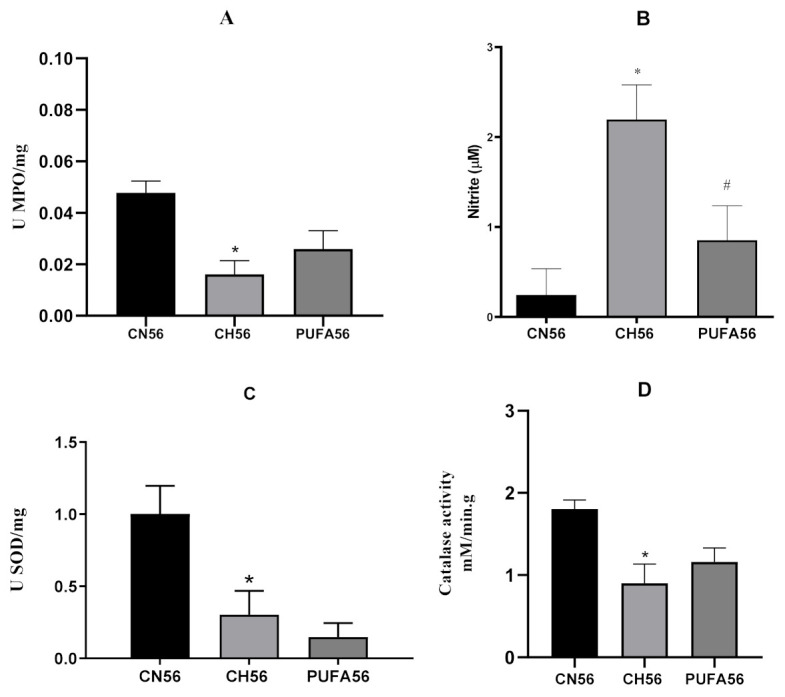
Effects of ω-3 PUFA-rich oil on myeloperoxidase activity (**A**), nitrite concentration (**B**), superoxide dismutase activity (**C**), and catalase activity (**D**) in rat aortic tissue homogenates from hypercholesterolemic rats after supplementation for 56 days. Mean ± SEM (standard error of the mean) of 8 animals/group. Unpaired Student’s *t*-test. * *p* < 0.05 vs. CN56; # *p* < 0,05 vs. CH56.

Measurements of myeloperoxidase (MPO), superoxide dismutase (SOD), and catalase (CAT) activity, as well as determination of nitrite (NO_2_^−^) concentration, were carried out in rat aortic homogenates. In the present study, decreases in MPO (Figure 7A), SOD (Figure 7C), and CAT (Figure 7D) activities in aortic homogenates from hypercholesterolemic rats (CH56 group) were observed when compared to the CN56 group (*p* < 0.05). However, supplementation with ω-3 PUFA-rich oil did not promote any increase in these activities.

On the other hand, the production of nitrite, an indicator of nitric oxide (NO) production, was measured by the Griess reaction, and its concentration was determined by interpolation with a NaNO_2_ calibration curve. Figure 6B shows a significant increase in nitrite concentration in aortic homogenates of hypercholesterolemic rats (CH56 group) when compared to those from rats in the CN56 group (*p* < 0.05). Interestingly, supplementation with ω-3 PUFA-rich oil promoted a marked decrease in nitrite concentration when compared with that in the CH56 group.

## 4. Discussion

The main finding of the present study was the beneficial effect promoted by ω-3 PUFAs on the cardiovascular morphology and vascular function of the aortas of animals with diet-induced hypercholesterolemia. Effects of treatment with ω-3 PUFA formulations on lipid profile have been evaluated in other studies. Evidence from clinical [29] and preclinical [30,31,32] studies has demonstrated that consumption of ω-3 PUFA decreases plasma TG and VLDL levels.

In the present work, treatment with ω-3 PUFA-rich oil did not result in alteration of the lipid profile of hypercholesterolemic rats, which was not unexpected, since there was no significant increase in TG levels. Besides, the effects of ω-3 PUFAs on cholesterol and lipoproteins seem to be controversial. Studies by Khandelwal et al., Tagawa et al., Eslick et al. pointed out a slight or even no effect of ω-3 PUFA supplementation on cholesterol and HDL and LDL lipoprotein levels [33,34,35]. On the other hand, [36] showed an increase in LDL and HDL levels after treatment with ω-3 PUFA. We believe that this may be a consequence of differences in experimental models, which comprise animal species, sex, age, and variability in the lipid profile, including the relatively low basal levels of serum TG obtained from diet, which aims to promote endothelial dysfunction secondary to hypercholesterolemia.

In hypercholesterolemic animals, an increase in blood glucose, an important cardiovascular risk factor, was observed (Table 2). In addition to changes in plasma lipids, hyperglycemia can induce endothelial cell dysfunction and functional abnormality of VSMCs through multiple pathways, such as increased oxidative stress, the release of reactive oxygen/nitrogen species, and increased iNOS expression [37,38]. As observed for the lipid profile, supplementation with ω-3 PUFA did not significantly change glucose levels.

The consumption of a hypercholesterolemic diet is related to increased hepatic lipid flow, which may result in the development of nonalcoholic fatty liver disease (NAFLD) [39], a pathology associated with an increased risk of CVD [40]. In PUFA56-group animals, a decrease in serum levels of liver damage markers (ALT, AST) was observed when compared with those in hypercholesterolemic animals (Table 2). Although a close association between NAFLD and coronary artery disease (CAD) is not yet clear, high risk of CVD has been related to increased oxidative stress, subclinical inflammation, and changes in lipid profile [41]. In a study by Godea et al., consumption of omega-3 PUFA slightly attenuated aortic changes and liver lipid accumulation in rats fed regular diets [42]. Okada et al. observed that supplementation with ω-3 PUFAs resulted in downregulation of hepatic lipogenesis through the inhibition of transcription factors associated with lipogenesis, increased expression of proteins related to lipid transport by HDL, and modulation of lipid insulin resistance, actions that together generated general benefits in lipid metabolism [43]. Thus, the improvement in liver function promoted by ω-3 PUFA-rich oil, possibly associated with the reduction in inflammation and liver lipids, may be positively correlated with vascular function.

There is a strong association between high plasma cholesterol and the incidence of atherosclerosis [44]. Aortic endothelial cells absorb and metabolize LDL and, when overloaded with intracellular cholesterol, generate cholesterol crystals (CC) that accumulate in the subendothelial layer after a short period of a lipid-rich diet, which precedes macrophage infiltration [45]. Then, in the process of atheroma formation, endothelial injury leads to increased permeability, leukocyte adhesions, and release of cytokines that attract monocytes to the tunica intima of arteries, where they differentiate into macrophages that accumulate lipids, becoming "foam cells’. Macrophages readily ingest oxidized LDL cholesterol. An increased serum LDL increases the oxidized LDL component, promoting the formation of atheromatous lesions [46,47] In the present study, aortic arches showed that the CH56 and PUFA56 groups had a slight pathological intimal thickening, with smooth muscle cell proliferation, lipid deposition, and foam cells in the aortic wall.

For this reason, the effects of supplementation with ω-3 PUFA-rich oil on the morphological changes identified in the myocardium and aortic arch of hypercholesterolemic rats were evaluated. Data obtained from cardiac tissue indicated that the initial stages of the healing process were first triggered in the PUFA56 group (Figure 3), suggesting that supplementation could improve hemodynamic parameters that support the repair process and consequently reverse the progression of the pathological changes in the cardiac tissue [48]. Likewise, treatment with ω-3 PUFA-rich oil promoted preservation of the tunica intima and structural reorganization of VSMCs and elastic fibers of the tunica media (Figure 4). Takashima et al. demonstrated that treatment with ω-3 PUFA decreased lipid deposition, macrophage accumulation, and the expression of inflammatory molecules in atherosclerotic plaques in the aortic root [49]. Mani et al. demonstrated that resolvin D1, the endogenous metabolite of docosahexaenoic acid (DHA), in addition to participating in the resolution of inflammation, protects the endothelial barrier against disturbances induced by cholesterol crystals [46].

Besides, it is well known that diet-induced hypercholesterolemia can result in vascular damage, especially in the endothelium, leading to impaired vascular relaxant responses and an increased response to vasoconstrictor agonists such as phenylephrine (PEN) [50,51,52]. Regarding the contractile response to FEN, vasoconstriction was potentiated in CH56 but not in CN56, showing that the consumption of hypercholesterolemic food for 16 weeks was effective in inducing functional damage to the endothelium. Phenylephrine is an adrenergic agonist that acts on specific receptors. In VSMCs, the binding of this agonist to α1-adrenergic receptors promotes vasoconstriction through the mobilization of intracellular and extracellular calcium, mainly through the generation of inositol-1,4,5-triphosphate (IP3) and D-1,2-diacylglycerol (DAG), respectively, with consequent activation of protein kinase C (PKC) [53,54]. Dora et al. demonstrated that the activation of adrenergic receptors in VSMCs, in addition to the direct vasoconstrictor effect, results in increases in NO production and release and the opening of K^+^ channels in the vascular endothelium. These actions occur because of the increase in intracellular Ca^2+^ concentration in ECs, which is due to a signaling process between VSMCs and ECs. This indirect effect of FEN on endothelium-derived vasorelaxation acts by modulating the contraction and explains the increase in the vasoconstrictor response by adrenergic stimulation in the absence of endothelium [55]. Interestingly, supplementation with ω-3 PUFA-rich oil abolished the increase in vascular reactivity when compared with CH56 group, indicating a role of this supplementation as a vascular protective, since reactivity to FEN was restored back to normal.

Regarding the role of endothelial function in vascular response, under physiological conditions, acetylcholine (ACh) induces an endothelium-dependent vasorelaxant response via NO release in response to vasoactive agonists. However, in the presence of significant endothelial dysfunction, NO release is reduced or abolished, decreasing the vasorelaxant response. After 56 days of treatment, animals supplemented with ω-3 PUFA-rich oil showed increased vasorelaxation (Emax = 74.20 ± 6.24%) induced by ACh when compared to those in the CH56 group (Emax = 61.73 ± 7.77%). Our results are consistent with other studies that have reported that PUFA ω-3 promoted improvement in endothelial function and increases in endothelium-dependent vasorelaxation [56,57,58,59,60,61].

Regarding the endothelium-independent vascular response, an increase in NPS-induced vasorelaxation was observed at concentrations of 3 × 10^−8^ M (51.52%) and 10^−8^ M (73.59%) in aortic preparations from the PUFA56 group when compared that in preparations from the CH56 group (23.05% and 43.47%). NPS is an inorganic hypotensive prodrug that works mainly as an endothelium-independent vasodilator. The direct release of NO inhibits guanylyl cyclase, leading to an increase in cGMP and PKG activation [62]. In their work, Zeiher et al. observed that when atherosclerosis was more advanced, the vasodilator response to nitroglycerin, a NO donor and smooth muscle relaxant, was significantly reduced, indicating an overall reduction in vasodilating capacity [63]. In our study, there was a difference in the response to the vasodilator NPS only at the lowest concentrations of NPS tested, showing that the relaxation capacity of CMLs to NO was largely preserved, even in hypercholesterolemic animals.

The mechanisms by which ω-3 PUFAs exert their effects are likely to be multiple and complex. Evidence has suggested the possibility of PUFA ω-3 supplementation altering EC membrane fluidity, resulting in potential effects on endothelial function. This event is related to changes in the composition of the membrane lipid bilayer with incorporation of these PUFAs into phospholipids, leading to the modulation of membrane-associated proteins and receptor activity and promoting increased synthesis and/or release of NO [56,64]. In addition, rapid and direct effects of these PUFAs include Ca^2+^-independent activation of eNOS; translocation and dissociation of caveolin-1 from eNOS in endothelial cells in situ [65]; and activation of TRPV4-type Ca^2+^-permeable ion channels in the endothelium, which improves flow-mediated vasodilation [66] as well as increased expression of eNOS and stimulation of NO production in ECs [59].

In order to assess the involvement of ω-3 PUFA-induced antioxidant mechanisms in vascular function, vascular reactivity was evaluated in the presence of pyrogallol, a superoxide anion donor. After incubation with pyrogallol, arteries from hypercholesterolemic animals showed a greater vasorelaxant effect in response to ACh when compared to control (CN56) and ω-3 PUFA-treated groups (Figure 6). Evidence has suggested that iNOS in disease is harmful because of its ability to produce excess NO [67]. A study by Hernanz et al. provided direct evidence that iNOS induction, by exposure to LPS, was associated with increased NO formation and norepinephrine hyporeactivity [68]. Furthermore, iNOS activity can lead to the formation of releasable NO stores, facilitating the removal of excess NO [69] in a way that alters the relationship between NO concentration and vascular reactivity. Such stocks are sensitive to ERO [70]. An increase in the formation of these species, such as what occurs with the use of a superoxide donor, would result in the release of NO from the stocks, making them bioavailable for action. In ApoE -/- mice, Pisaniello et al. observed reduced aortic expression of inflammatory chemokines and adhesion molecules after treatment with ω-3 PUFA [71]. These findings possibly justify the absence of pronounced relaxation in hypercholesterolemic animals in nontreated arteries with pyrogallol and an increase in this relaxation when these arteries are exposed to pyrogallol.

MPO activity was evaluated in aortic homogenate as a possible biomarker of leukocyte activity. Our results revealed decreased vascular MPO activity in hypercholesterolemic (CH56) rats compared to CN56 rats (Figure 7A). There have been many publications in which MPO seemed to have a prooxidant role, leading to atherogenesis. On the other hand, there have been experimental data showing an unexpected protective role of MPO in atherosclerosis. In a hypercholesterolemic murine model, Brennan et al. described increased atherosclerosis in MPO-deficient mice [71]. Békési et al. [72] revealed that atherosclerotic structural alterations of the aorta in animals treated with MPO and cholesterol inhibitors may be related to a decrease in peroxidase activity. Our results may, in part, support the findings of Pradhan-Palikhe et al. (2010) and Kubala et al., who reported that MPO expression and levels were not elevated in stable arterial disease. Taken together, these studies indicate that increased MPO activity is not necessarily characteristic of asymptomatic arterial disease, such as the vascular dysfunction reported in the present study [73,74].

Nitrite (NO_2_^−^) is an indicator of NO production that can be at higher levels during inflammatory conditions [75]. In the present study, an increase in NO_2_^−^ concentration in aortic homogenates was observed in hypercholesterolemic rats (Figure 7B), which corroborated the hypothesis about greater production of NO due to increased iNOS activity in this physiopathological condition. Lu et al. demonstrated that EPA and DHA effectively inhibited interferon-γ (IFN-γ)-induced iNOS and COX-2 expression in microglial and neuronal cells, as well as antagonizing interferon-γ-induced NO production [76]. A similar result was observed in LPS-activated murine macrophages, where DPA reduced the mRNA expression of proinflammatory factors such as iNOS and COX-2 [77]. In ApoE -/- mice, Pisaniello et. al. observed reduced aortic expression of inflammatory chemokines and adhesion molecules after treatment with ω-3 PUFA [78]. Such evidence points to an important modulation of the inflammatory response by ω-3 PUFA, which possibly correlates with the vascular effects observed in this study.

The activity of SOD and CAT enzymes in aortic artery homogenate was observed to assess whether findings in vascular reactivity were associated with the activity of these antioxidant enzymes and with the vascular concentration of O_2_^−^ and H_2_O_2_. A decrease in SOD activity, as seen in the CH56 group, reduces O_2_^−^ dismutation. In the vessel, changes in O_2_^−^ levels have been shown to modulate vascular tone, gene expression, inflammation, cell growth, signaling, and apoptosis [79]. In addition, O_2_^−^ is a precursor to other ROS, including H_2_O_2_ and peroxynitrite (ONOO^−^). ONOO^−^, formed from O_2_^−^ and NO produced by iNOS, is involved in cytotoxicity related to excess NO and vascular hyporeactivity [80]. In the present study, no significant difference was observed in the SOD activity of the ω-3 PUFA-treated animals (Figure 7C).

Likewise, a decrease in CAT activity was observed in the aortic homogenates of hypercholesterolemic animals, which results in accumulation of H_2_O_2_ in vascular tissue, which may be involved in the vascular response observed in the CH56 group after incubation with pyrogallol. H_2_O_2_ induces vasodilation in different vascular beds, such as mesenteric, coronary, or pulmonary [80,81,82,83,84,85]. In smooth muscle, H_2_O_2_ reacts with NO, generating nitrite and hydroxyl anions, which also lead to vasodilation [85,86]. In the present study, no significant difference was observed in the SOD activity of the ω-3 PUFA-treated animals (Figure 7D).

A limiting factor comprises the coefficients of variation (%CV) of protocols regarding biochemical and antioxidant assessment. A validation study of those experimental protocols and the calculation of %CV using gold standards is out of the scope of this work. Furthermore, differences among idiosyncratic responsiveness to hypercholesterolemic diet in rats could constitute a limiting factor for obtention of suitable values of %CV. Therefore, standard deviation and error are usual parameters to assess statistical significance. Those protocols and reference values have been widely reported and reproduced in several previous studies, which have supported the reproducibility and reliability of the present findings.

## 5. Conclusions

A favorable effect of ω-3 PUFA-rich oil supplementation was documented in terms of preserving the morphology and vascular reactivity of adult male hypercholesterolemic rats, repairing cardiac tissue damage, decreasing phenylephrine-induced vasoconstrictor hyperreactivity, and increasing vasorelaxation induced by ACh, suggesting an improvement in vascular endothelial function. In addition, ω-3 PUFA-rich oil contributed to a reduction in liver function biomarkers. Our results suggest that the vascular effects of ω-3 PUFAs involve improvement in endothelial function and maintenance of the cellular structure of the aortic tissue affected by hypercholesterolemia. These findings provide relevant support for the benefits of ω-3 PUFA supplementation on cardiovascular health.

## Figures and Tables

**Table 1:** Fatty acid composition in ω-3 polyunsaturated fatty acid-rich oil capsules determined by GC–MS.

Peak	Systematic Name	Fatty Acid	Classification *	(M^+^)	Retention Time (min)	Relative Intensity (%)	
1	Tetradecanoic acid	C14:0	SFA	242	23.881	6.88	33.57
2	Pentadecanoic acid	C15:0	SFA	256	25.776	0.47	
6	Hexadecanoic acid	C16:0	SFA	270	27.598	21.75	
11	Octadecanoic acid	C18:0	SFA	298	30.956	4.14	
16	Eicosanoic acid	C20:0	SFA	326	34.033	0.33	
5	Hexadec-9-enoic acid	C16:1	MUFA	268	27.232	11.85	30.28
9	Octadec-9-enoic acid	C18:1	MUFA	296	30.559	12.39	
10	Octadec-11-enoic acid	C18:1	MUFA	296	30.644	3.05	
15	Eicosa-11-enoic acid	C20:1	MUFA	324	33.669	1.33	
20	Docosa-13-enoic acid	C22:1	MUFA	354	36.485	1.66	
7	Octadec-6,9,12,15-tetraenoic acid	C18:4 ω3	PUFA	290	30.294	2.94	31.01
4	Hexadec-7,10,13-trienoic acid	C16:3 ω3	PUFA	264	27.009	1.27	
13	Eicosa-5,8,11,14,17-pentaenoic acid (EPA)	C20:5 ω3	PUFA	316	33.161	16.39	
14	Eicosa-11,14,17-trienoic acid	C20:3 ω3	PUFA	320	33.419	0.70	
17	Docosa-7,10,13,16,19-pentaenoic acid	C22:5 ω3	PUFA	344	34.658	0.45	
18	Docosa-4,7,10,13,16,19-hexaenoic acid (DHA)	C22:6 ω3	PUFA	342	35.867	9.26	
8	Octadec-9,12-dienoic acid	C18:2 ω6	PUFA	294	30.451	1.21	3.61
12	Eicosa-5,8,11,14-tetraenoic acid	C20:4 ω6	PUFA	318	33.050	0.80	
19	Docosa-13,16-dienoic acid	C22:2 ω6	PUFA	352	36.056	1.60	
3	N.I.	—		—	26.897	1.53	1.53

* MUFA—monounsaturated fatty acids; PUFA—polyunsaturated fatty acids; SFA—saturated fatty acids; ω3—omega-3; ω6—omega 6; NI—non-identified.

**Table 2:** Biochemical parameters of animals subjected to experimental hypercholesterolemia induction and treated with ω-3 PUFA-rich oil. Mean ± SEM (standard error of the mean) of 8 animals/group. Unpaired Student’s *t*-test.

Serum Parameters	Experimental Groups		
	CN56	CH56	PUFA56
Total cholesterol (mg/dL)	78.00 ± 5.28	122.3 ± 12.45 *	140.9 ± 24.30
Triglycerides (mg/dL)	62.50 ± 9.53	51.73 ± 8.58	65.00 ± 13.26
LDL (mg/dL)	36.00 ± 3.763	78.84 ± 0.97 *	109.5 ± 16.65
HDL (mg/dL)	33.64 ± 2.58	36.40 ± 2.34	34.43 ± 3.26
VLDL (mg/dL)	12.50 ± 1.90	9.52 ± 0.92	13.00 ± 2.65
Glucose (g/dL)	141.6 ± 7.61	180.5 ± 13.57 *	161.1 ± 12.03
Albumin (g/dL)	2.066 ± 0.065	2.090 ± 0.109	1.498 ± 0.197 #
Total proteins (g/dL)	7.366 ± 0.187	8.039 ± 0.162 *	6.619 ± 0.759 #
ALT (U/L)	60.00 ± 4.251	79.42 ± 5.103 *	53.57 ± 7.054 #
AST (U/L)	131.9 ± 5.019	136.5 ± 9.894	97.88 ± 12.79 #

Legend: * *p* < 0.05 vs. CN56; # *p* < 0.05 vs. CH. PUFA—polyunsaturated fatty acids; LDL—low density lipoprotein; HDL—high-density lipoprotein; VLDL—very low-density lipoprotein.

## Supplementary Materials

The following are available online at https://www.mdpi.com/article/10.3390/biology11020202/s1, Figure S1: Photomicrographs of aorta from CH56 group. Masson’s trichrome stain (scale bar: 20 µm and 60 µm). A: This cross-section of the aorta shows some foam cells and lipid deposition in the middle layer (tunica media), with diffusely moderate deposition of collagen fibers (*) and fibrin network (arrow) adherent to the endothelium (tunica intima). B: Detail of A showing disarrangement of the elastic fibers of the intermediate layer, foam cell (arrowhead), and slightly thickened subendothelial connective tissue (arrow).

## References

[1] World Health Organization (WHO) (2017). Cardiovacular Diseases (CVDs). Fact Sheet nº 31. https://www.who.int/news-room/fact-sheets/detail/cardiovascular-diseases-(cvds)/.

[2] Manach C., Mazur A., Scalbert A. (2006). Polyphenols and prevention of cardiovascular diseases. Curr. Opin. Lipidol..

[3] De Pinho R.A., de Araújo M.C., Ghisi G.L.D.M., Benetti M. (2010). Doença arterial coronariana, exercício físico e estresse oxidativo. Arq. Bras. Cardiol..

[4] Libby P. (2002). Inflammation in atherosclerosis. Nature.

[5] Lusis A.J. (2000). Atherosclerosis. Nature.

[6] (2020). Carotid Artery Stenosis. In StatPearls Publishing. https://www.ncbi.nlm.nih.gov/books/NBK442025/.

[7] Kobiyama K., Ley K. (2018). Atherosclerosis. Circ. Res..

[8] Napoli C., Paternò R., Faraci F.M., Taguchi H., Postiglione A., Heistad D.D. (1997). Mildly oxidized low-density lipoprotein impairs responses of carotid but not basilar artery in rabbits. Stroke.

[9] Santos R.D., Gagliardi A.C.M., Xavier H.T., Magnoni C.D., Cassani R., Lottenberg A.M.P., Filho A.C., Araújo D., Cesena F., Alves R. (2013). I Diretriz sobre o consumo de gorduras e saúde cardiovascular. Arq. Bras. Cardiol..

[10] Torres N., Guevara-Cruz M., Velázquez-Villegas L.A., Tovar A.R. (2015). Nutrition and Atherosclerosis. Arch. Med. Res..

[11] Bang H.O., Dyerberg J., Hjøorne N. (1976). The composition of food consumed by Greenland Eskimos. Acta Med. Scand..

[12] Dyerberg J., Bang H.O., Stoffersen E., Moncada S., Vane J.R. (1978). Eicosapentaenoic acid and prevention of thrombosis and atherosclerosis?. Lancet.

[13] Mori T.A. (2014). Omega-3 fatty acids and cardiovascular disease: Epidemiology and effects on cardiometabolic risk factors. Food Funct..

[14] Feuchtner G., Langer C., Barbieri F., Beyer C., Dichtl W., Friedrich G., Schgoer W., Widmann G., Plank F. (2021). The effect of omega-3 fatty acids on coronary atherosclerosis quantified by coronary computed tomography angiography. Clin. Nutr..

[15] Abriz A.E., Rahbarghazi R., Nourazarian A., Avci Ç.B., Mahboob S.A., Rahnema M., Araghi A., Heidarzadeh M. (2021). Effect of docosahexaenoic acid plus insulin on atherosclerotic human endothelial cells. J. Inflamm..

[16] Chowdhury R., Stevens S., Gorman D., Pan A., Warnakula S., Chowdhury S., Ward H., Johnson L., Crowe F., Hu F.B. (2012). Association between fish consumption, long chain omega 3 fatty acids, and risk of cerebrovascular disease: Systematic review and meta-analysis. BMJ.

[17] Hartman L., Lago R.C. (1973). Rapid preparation of fatty acid methyl esters from lipids. Lab. Pract..

[18] Azuma K., Nagae T., Nagai T., Izawa H., Morimoto M., Murahata Y., Osaki T., Tsuka T., Imagawa T., Ito N. (2015). Effects of Surface-Deacetylated Chitin Nanofibers in an Experimental Model of Hypercholesterolemia. Int. J. Mol. Sci..

[19] Baracho N.C.D.V., Nunes L.A.S., De Paula e Silva K.T., Marques T.F., Dos Santos A.L.R., Marcelino A.R. (2014). Desenvolvimento de um Modelo Experimental de Dislipidemia de Baixo Custo. Rev. Cienc. Saude.

[20] Guerra R.L., Prado W.L., Cheik N.C., Viana F.P., Botero J.P., Vendramini R.C., Carlos I.Z., Rossi E.A., Dâmaso A.R. (2007). Effects of 2 or 5 consecutive exercise days on adipocyte area and lipid parameters in Wistar rats. Lipids Health Dis..

[21] Nisar J., Mustafa I., Anwar H., Sohail M.U., Hussain G., Ullah M.I., Faisal M.N., Bukhari S.A., Basit A. (2017). Shiitake Culinary-Medicinal Mushroom, Lentinus edodes (Agaricomycetes): A Species with Antioxidant, Immunomodulatory, and Hepatoprotective Activities in Hypercholesterolemic Rats. Int. J. Med. Mushrooms.

[22] Saravanan S., Srikumar R., Manikandan S., Jeya Parthasarathy N., Sheela Devi R. (2007). Hypolipidemic Effect of Triphala in Experimentally Induced Hypercholesteremic Rats. Yakugaku Zasshi.

[23] Friedewald W.T., Levy R.I., Fredrickson D.S. (1972). Estimation of the concentration of low-density lipoprotein cholesterol in plasma, without use of the preparative ultracentrifuge. Clin. Chem..

[24] Arcanjo D.D.R., Vasconcelos A.G., Comerma-Steffensen S.G., Jesus J.R., Silva L.P., Pires O.R., Costa-Neto C.M., Oliveira E.B., Migliolo L., Franco O.L. (2015). A Novel Vasoactive Proline-Rich Oligopeptide from the Skin Secretion of the Frog *Brachycephalus ephippium*. PLoS ONE.

[25] Bradley P.P., Priebat D.A., Christensen R.D., Rothstein G. (1982). Measurement of cutaneous inflammation: Estimation of neutrophil content with an enzyme marker. J. Investig. Dermatol..

[26] Green L.C., Wagner D.A., Glogowski J., Skipper P.L., Wishnok J.S., Tannenbaum S.R. (1982). Analysis of nitrate, nitrite, and [15N]nitrate in biological fluids. Anal. Biochem..

[27] Jascolka T.L. (2010). Efeitos do Quefir no Perfil Lipídico, Estresse Oxidativo e Aterosclerose de Camundongos Deficientes em Apolipoproteína, E. Master’s Thesis.

[28] Beutler E. (1975). Metabolismo das Células Vermelhas: Um Manual de Métodos Bioquímicos.

[29] Luzia L.A., Aldrighi J.M., Damasceno NR T., Sampaio G.R., Soares RA M., Silva I.T., de Queiroz Mello A.P., Carioca A.A.F., da Silva Torres E.A.F. (2015). Fish Oil and Vitamin E Change Lipid Profiles and Anti-Ldl-Antibodies in Two Different Ethnic Groups of Women Transitioning through Menopause. Nutr. Hosp..

[30] Chang C.L., Torrejon C., Jung U.J., Graf K., Deckelbaum R.J. (2014). Incremental replacement of saturated fats by n-3 fatty acids in high-fat, high-cholesterol diets reduces elevated plasma lipid levels and arterial lipoprotein lipase, macrophages and atherosclerosis in LDLR-/-mice. Atherosclerosis.

[31] Malinska H., Hüttl M., Oliyarnyk O., Bratova M., Kazdova L. (2015). Conjugated linoleic acid reduces visceral and ectopic lipid accumulation and insulin resistance in chronic severe hypertriacylglycerolemia. Nutrition.

[32] Ramaiyan B., Bettadahalli S., Talahalli R.R. (2016). Dietary omega-3 but not omega-6 fatty acids down-regulate maternal dyslipidemia induced oxidative stress: A three generation study in rats. Biochem. Biophys. Res. Commun..

[33] Khandelwal S., Shidhaye R., Demonty I., Lakshmy R., Gupta R., Prabhakaran D., Reddy S. (2013). Impact of omega-3 fatty acids and/or plant sterol supplementation on non-HDL cholesterol levels of dyslipidemic Indian adults. J. Funct. Foods.

[34] Tagawa T., Hirooka Y., Shimokawa H., Hironaga K., Sakai K., Oyama J.-I., Takeshita A. (2002). Long-Term Treatment with Eicosapentaenoic Acid Improves Exercise-Induced Vasodilation in Patients with Coronary Artery Disease. Hypertens. Res..

[35] Eslick G.D., Howe P.R., Smith C., Priest R., Bensoussan A. (2009). Benefits of fish oil supplementation in hyperlipidemia: A systematic review and meta-analysis. Int. J. Cardiol..

[36] Maki K.C., Van Elswyk M.E., McCarthy D., Hess S.P., Veith P.E., Bell M., Subbaiah P., Davidson M.H. (2005). Lipid responses to a dietary docosahexaenoic acid supplement in men and women with below average levels of high density lipoprotein cholesterol. J. Am. Coll. Nutr..

[37] Kaplan M., Aviram M., Hayek T. (2012). Oxidative stress and macrophage foam cell formation during diabetes mellitus-induced atherogenesis: Role of insulin therapy. Pharmacol. Ther..

[38] Li T., Yang G.M., Zhu Y., Wu Y., Chen X.Y., Lan D., Tian K.L., Liu L.M. (2015). Diabetes and hyperlipidemia induce dysfunction of VSMCs: Contribution of the metabolic inflammation/miRNA pathway. Am. J. Physiol. Endocrinol. Metab..

[39] Ahmed U., Redgrave T.G., Oates P.S. (2009). Effect of dietary fat to produce non-alcoholic fatty liver in the rat. J. Gastroenterol. Hepatol..

[40] Francque S.M. (2014). The Role of Non-alcoholic Fatty Liver Disease in Cardiovascular Disease. Eur. Cardiol..

[41] Efe D., Aygün F. (2013). Assessment of the Relationship between Non-Alcoholic Fatty Liver Disease and CAD using MSCT. Arq. Bras. Cardiol..

[42] Godea Lupei S., Ciubotariu D., Danciu M., Lupușoru R.V., Ghiciuc C.M., Cernescu I., Gheţu N., Lupei M., Lupușoru C.E. (2020). Improvement in serum lipids and liver morphology after supplementation of the diet with fish oil is more evident under regular feeding conditions than under high-fat or mixed diets in rats. Lipids Health Dis..

[43] Okada L.S.D.R.R., Oliveira C.P., Stefano J.T., Nogueira M.A., da Silva I.D.C.G., Cordeiro F., Alves V.A.F., Torrinhas R.S., Carrilho F.J., Puri P. (2018). Omega-3 PUFA modulate lipogenesis, ER stress, and mitochondrial dysfunction markers in NASH-Proteomic and lipidomic insight. Clin. Nutr..

[44] Rafieian-Kopaei M., Setorki M., Doudi M., Baradaran A., Nasri H. (2014). Atherosclerosis: Process, indicators, risk factors and new hopes. Int. J. Prev. Med..

[45] Baumer Y., Mccurdy S., Weatherby T.M., Mehta N.N., Halbherr S., Halbherr P., Yamazaki N., Boisvert W.A. (2017). Hyperlipidemia-induced cholesterol crystal production by endothelial cells promotes atherogenesis. Nat. Commun..

[46] Mani A.M., Chattopadhyay R., Singh N.K., Rao G.N. (2018). Cholesterol crystals increase vascular permeability by inactivating SHP2 and disrupting adherens junctions. Free Radic. Biol. Med..

[47] Ansari M.A., Iqubal A., Ekbbal R., Haque S.E. (2019). Effects of nimodipine, vinpocetine and their combination on isoproterenol-induced myocardial infarction in rats. Biomed. Pharmacother..

[48] Virmani R., Kolodgie F.D., Burke A.P., Farb A., Schwartz S.M. (2000). Lessons from Sudden Coronary Death. Arterioscler. Thromb. Vasc. Biol..

[49] Takashima A., Fukuda D., Tanaka K., Higashikuni Y., Hirata Y., Nishimoto S., Yagi S., Yamada H., Soeki T., Wakatsuki T. (2016). Combination of n-3 polyunsaturated fatty acids reduces atherogenesis in apolipoprotein E-deficient mice by inhibiting macrophage activation. Atherosclerosis.

[50] Da Motta N.A.V., Kümmerle A.E., Marostica E., Dos Santos C.F., Fraga C.A.M., Barreiro E.J., De Miranda A.L.P., De Brito F.C.F. (2013). Anti-atherogenic Effects of a New Thienylacylhydrazone Derivative, LASSBio-788, in Rats Fed a Hypercholesterolemic Diet. J. Pharmacol. Sci..

[51] Rahman E., Donia S.S., Naguib Y.M. (2013). Garlic improves altered vascular reactivity and plasma lipids in high cholesterol-fed rats. Menoufia Med. J..

[52] Silva N.L.C., Motta N.A.V., Soares M.A., Araujo O.M.O., Espíndola L.C.P., Colombo A.P.V., Lopes R.T., Brito F.C.F., Miranda A.L.P., Tributino J.L.M. (2020). Periodontal status, vascular reactivity, and platelet aggregation changes in rats submitted to hypercholesterolemic diet and periodontitis. J. Periodontal Res..

[53] Minneman K.P. (1988). Alpha 1-adrenergic receptor subtypes, inositol phosphates, and sources of cell Ca^2+^. Pharmacol. Rev..

[54] Pan B.X., Zhao G.L., Huang X.L., Jin J.Q., Zhao K.S. (2004). Peroxynitrite induces arteriolar smooth muscle cells membrane hyperpolarization with arteriolar hyporeactivity in rats. Life Sci..

[55] Dora K.A., Hinton J.M., Walker S.D., Garland C.J. (2000). An indirect influence of phenylephrine on the release of endothelium-derived vasodilators in rat small mesenteric artery. Br. J. Pharmacol..

[56] Goodfellow J., Bellamy M.F., Ramsey M.W., Jones C.J., Lewis M.J. (2000). Dietary supplementation with marine omega-3 fatty acids improve systemic large artery endothelial function in subjects with hypercholesterolemia. J. Am. Coll. Cardiol..

[57] Tousoulis D., Plastiras A., Siasos G., Oikonomou E., Verveniotis A., Kokkou E., Maniatis K., Gouliopoulos N., Miliou A., Paraskevopoulos T. (2014). Omega-3 PUFAs improved endothelial function and arterial stiffness with a parallel antiinflammatory effect in adults with metabolic syndrome. Atherosclerosis.

[58] Zanetti M., Grillo A., Losurdo P., Panizon E., Mearelli F., Cattin L., Barazzoni R., Carretta R. (2015). Omega-3 Polyunsaturated Fatty Acids: Structural and Functional Effects on the Vascular Wall. Biomed. Res. Int..

[59] Zanetti M., Gortan Cappellari G., Barbetta D., Semolic A., Barazzoni R. (2017). Omega 3 Polyunsaturated Fatty Acids Improve Endothelial Dysfunction in Chronic Renal Failure: Role of eNOS Activation and of Oxidative Stress. Nutrients.

[60] Zgheel F., Perrier S., Remila L., Houngue U., Mazzucotelli J.P., Morel O., Auger C., Schini-Kerth V.B. (2019). EPA:DHA 6:1 is a superior omega-3 PUFAs formulation attenuating platelets-induced contractile responses in porcine coronary and human internal mammary artery by targeting the serotonin pathway via an increased endothelial formation of nitric oxide. Eur. J. Pharmacol..

[61] Farooq M.A., Gaertner S., Amoura L., Niazi Z.R., Park S.H., Qureshi A.W., Oak M.H., Toti F., Schini-Kerth V.B., Auger C. (2020). Intake of omega-3 formulation EPA:DHA 6:1 by old rats for 2 weeks improved endothelium-dependent relaxations and normalized the expression level of ACE/AT1R/NADPH oxidase and the formation of ROS in the mesenteric artery. Biochem. Pharmacol..

[62] Ranadive S.M., Eugene A.R., Dillon G., Nicholson W.T., Joyner M.J. (2017). Comparison of the vasodilatory effects of sodium nitroprusside vs. nitroglycerin. J. Appl. Physiol..

[63] Zeiher A.M., Drexler H., Wollschläger H., Just H. (1991). Modulation of coronary vasomotor tone in humans. Progressive endothelial dysfunction with different early stages of coronary atherosclerosis. Circulation.

[64] Chen H., Li D., Chen J., Roberts G.J., Saldeen T., Mehta J.L. (2003). EPA and DHA attenuate ox-LDL-induced expression of adhesion molecules in human coronary artery endothelial cells via protein kinase B pathway. J. Mol. Cell. Cardiol..

[65] Omura M., Kobayashi S., Mizukami Y., Mogami K., Todoroki-Ikeda N., Miyake T., Matsuzaki M. (2001). Eicosapentaenoic acid (EPA) induces Ca2+-independent activation and translocation of endothelial nitric oxide synthase and endothelium-dependent vasorelaxation. FEBS Lett..

[66] Zhu Y., Wen L., Wang S., Zhang K., Cui Y., Zhang C., Feng L., Yu F., Chen Y., Wang R. (2021). Omega-3 fatty acids improve flow-induced vasodilation by enhancing TRPV4 in arteries from diet-induced obese mice. Cardiovasc. Res..

[67] Lind M., Hayes A., Caprnda M., Petrovic D., Rodrigo L., Kruzliak P., Zulli A. (2017). Inducible nitric oxide synthase: Good or bad?. Biomed. Pharmacother..

[68] Hernanz R., Alonso M.J., Zibrandtsen H., Alvarez Y., Salaices M., Simonsen U. (2004). Measurements of nitric oxide concentration and hyporeactivity in rat superior mesenteric artery exposed to endotoxin. Cardiovasc. Res..

[69] Kleschyov A.L., Muller B., Keravis T., Stoeckel M.E., Stoclet J.C. (2000). Adventitia-derived nitric oxide in rat aortas exposed to endotoxin: Cell origin and functional consequences. Am. J. Physiol. Heart Circ. Physiol..

[70] Muller B., Kleschyov A.L., Alencar J.L., Vanin A., Stoclet J.-C. (2002). Nitric Oxide Transport and Storage in the Cardiovascular System. Ann. N. Y. Acad. Sci..

[71] Brennan M.-L., Anderson M.M., Shih D.M., Qu X.-D., Wang X., Mehta A.C., Lim L.L., Shi W., Hazen S.L., Jacob J.S. (2001). Increased atherosclerosis in myeloperoxidase-deficient mice. J. Clin. Investig..

[72] Békési G., Heinle H., Kakucs R., Pázmány T., Szombath D., Dinya M., Tulassay Z., Fehér J., Rácz K., Székács B. (2005). Effect of inhibitors of myeloperoxidase on the development of aortic atherosclerosis in an animal model. Exp. Gerontol..

[73] Pradhan-Palikhe P., Vikatmaa P., Lajunen T., Palikhe A., Lepäntalo M., Tervahartiala T., Salo T., Saikku P., Leinonen M., Pussinen P.J. (2010). Elevated MMP-8 and Decreased Myeloperoxidase Concentrations Associate Significantly with the Risk for Peripheral Atherosclerosis Disease and Abdominal Aortic Aneurysm1. Scand. J. Immunol..

[74] Kubala L., Lu G., Baldus S., Berglund L., Eiserich J.P. (2008). Plasma levels of myeloperoxidase are not elevated in patients with stable coronary artery disease. Clin. Chim. Acta.

[75] Calcerrada P., Peluffo G., Radi R. (2011). Nitric oxide-derived oxidants with a focus on peroxynitrite: Molecular targets, cellular responses and therapeutic implications. Curr. Pharm. Des..

[76] Lu D.-Y., Tsao Y.-Y., Leung Y.-M., Su K.-P. (2010). Docosahexaenoic Acid Suppresses Neuroinflammatory Responses and Induces Heme Oxygenase-1 Expression in BV-2 Microglia: Implications of Antidepressant Effects for Omega-3 Fatty Acids. Neuropsychopharmacology.

[77] Tian Y., Katsuki A., Romanazzi D., Miller M.R., Adams S.L., Miyashita K., Hosokawa M. (2017). Docosapentaenoic Acid (22:5n-3) Downregulates mRNA Expression of Pro-inflammatory Factors in LPS-activated Murine Macrophage Like RAW264.7 Cells. J. Oleo Sci..

[78] Pisaniello A.D., Psaltis P.J., King P.M., Liu G., Gibson R.A., Tan J.T., Duong M., Nguyen T., Bursill C.A., Worthley M.I. (2021). Omega-3 fatty acids ameliorate vascular inflammation: A rationale for their atheroprotective effects. Atherosclerosis.

[79] Costa T.J., Barros P.R., Arce C., Santos J.D., da Silva-Neto J., Egea G., Dantas A.P., Tostes R.C., Jiménez-Altayó F. (2020). The homeostatic role of hydrogen peroxide, superoxide anion and nitric oxide in the vasculature. Free Radic. Biol. Med..

[80] Zhang M.L., Zheng B., Tong F., Yang Z., Wang Z.B., Yang B.M., Sun Y., Zhang X.H., Zhao Y.L., Wen J.K. (2017). iNOS-derived peroxynitrite mediates high glucose-induced inflammatory gene expression in vascular smooth muscle cells through promoting KLF5 expression and nitration. Biochim. Biophys. Acta Mol. Basis Dis..

[81] Fujimoto S., Asano T., Sakai M., Sakurai K., Takagi D., Yoshimoto N., Itoh T. (2001). Mechanisms of hydrogen peroxide-induced relaxation in rabbit mesenteric small artery. Eur. J. Pharmacol..

[82] Gao Y.-J., Hirota S., Zhang D.-W., Janssen L.J., Lee R.M.K.W. (2003). Mechanisms of hydrogen-peroxide-induced biphasic response in rat mesenteric artery. Br. J. Pharmacol..

[83] Sato A., Sakuma I., Gutterman D.D. (2003). Mechanism of dilation to reactive oxygen species in human coronary arterioles. Am. J. Physiol. Heart Circ. Physiol..

[84] Bretón-Romero R., Lamas S. (2014). Hydrogen peroxide signaling in vascular endothelial cells. Redox Biol..

[85] Prasad K., Bharadwaj L.A. (1996). Hydroxyl radical—A mediator of acetylcholine-induced vascular relaxation. J. Moll. Cell. Cardiol..

[86] Farias-Eisner R., Chaudhuri G., Aeberhard E., Fukuto J.M. (1996). The Chemistry and Tumoricidal Activity of Nitric Oxide/Hydrogen Peroxide and the Implications to Cell Resistance/Susceptibility. J. Biol. Chem..

