# Supplementary figures and images for: Effects of ω-3 PUFA-Rich Oil Supplementation on Cardiovascular Morphology and Aortic Vascular Reactivity of Adult Male Rats Submitted to an Hypercholesterolemic Diet
Source: Biology (Basel). 2022 Jan 27;11(2):202. doi: 10.3390/biology11020202 (PMC8869584; doi:10.3390/biology11020202)

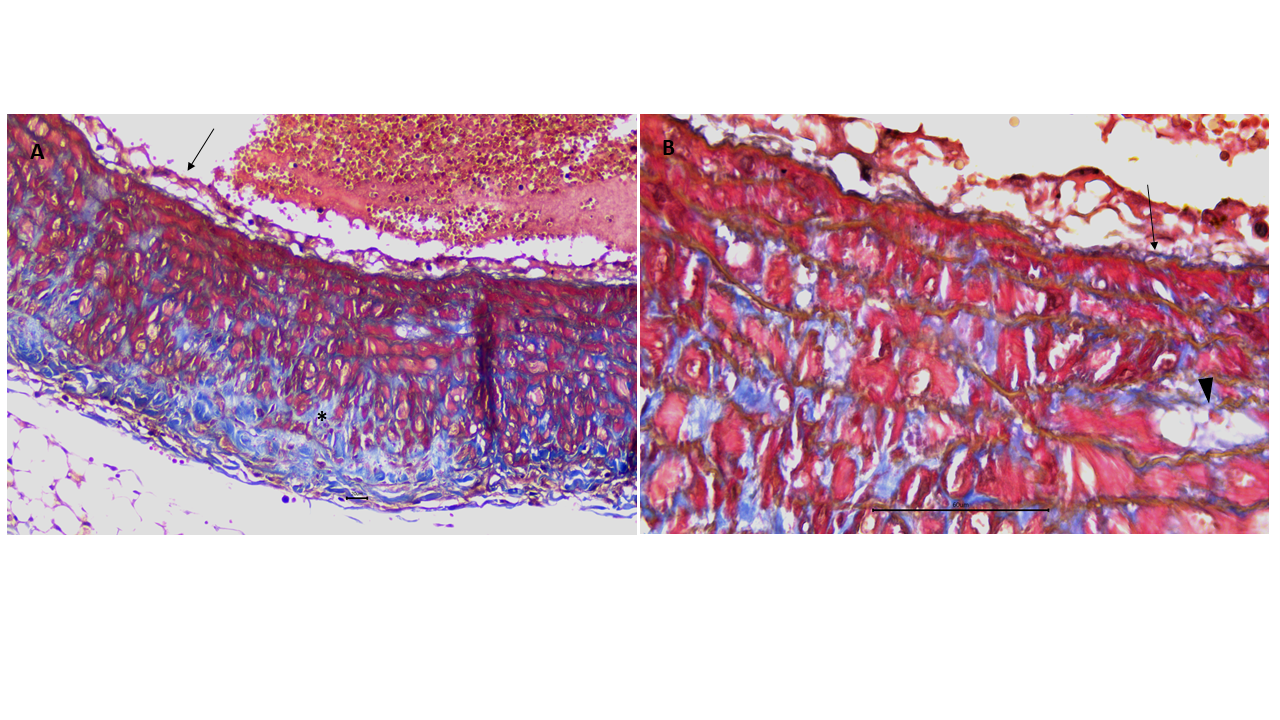

Supplement: Supplementary file 1 [file biology-11-00202-s001.zip › biology-1521357-supplementary.tiff]
